# Biomechanical feedback and feedforward responses during perturbed running in asymptomatic individuals

**DOI:** 10.3389/fspor.2024.1403770

**Published:** 2024-11-22

**Authors:** Mina Khajooei, Andrew Quarmby, Frank Mayer, Tilman Engel

**Affiliations:** Sports Medicine and Sports Orthopaedics, University of Potsdam, University Outpatient Clinic, Potsdam, Germany

**Keywords:** feedback, feedforward, motor control, stumbling, running

## Abstract

Assessment of biomechanical features whilst running on an uneven terrain plays an important role in identifying running-related injury mechanisms. However, feedback and feedforward motor responses and adaptations, an important component of gait retraining and injury rehabilitation programs, have been less investigated during running. Therefore, the current study assessed the whole-session responses and within-session adaptation mechanisms during perturbed running. Twenty three individuals performed an eight-minute perturbed treadmill running protocol with one-sided decelerative belt perturbations. Joint angle curves and muscle activity amplitudes were analysed throughout the running cycle, in both the perturbed and contralateral leg. For the whole-session responses, the average of 10 consecutive strides during the baseline trial and all perturbed strides from the perturbed running trial were compared. To assess within-session adaptation, the first perturbation was compared to the average of the last three perturbations. Data were analysed with one-dimensional statistical parametric mapping of Paired *t*-tests to assess responses and adaptations to the perturbations (*P* < 0.025). Regarding whole-session responses (baseline vs. perturbations), statistically significant feedback (after perturbation) responses were detected in most measured joint angles and muscle activity of both perturbed and contralateral legs. Feedforward (before perturbation) responses for whole-session comparison were detected for most joint angles in the contralateral leg and only hip flexion in the perturbed leg. Feedforward muscle activities of whole-session responses were different in the biceps femoris, semitendinosus, and erector spinae of the perturbed leg, and the soleus of the contralateral leg. Regarding within-session (first vs. last three perturbations) adaptation, feedback adaptations included statistically significant changes in ankle, knee, and hip movements, and muscle activities in the perturbed leg, while the contralateral leg showed less adaptation. No significant feedforward within-session adaptations were observed in the perturbed leg, but the contralateral leg showed changes in ankle dorsiflexion, soleus activity, and erector spinae activity. Findings suggest that participants compensated perturbations during running by modifying muscle activities and movement patterns, primarily through feedback mechanisms in the perturbed leg, with limited feedforward adaptations. The current protocol may present a viable approach for testing and training postural control during running.

## 1 Introduction

Trail running has become increasingly popular among professional and recreational runners in recent years, but unpredictable changes in terrain that is characteristic of this activity can place extra stress on joints and muscles, potentially leading to running-related injuries (RRIs) (1–3). To identify injury mechanisms and improve performance, it is important to assess the biomechanical features of running when exposed to unexpected perturbations (2–4).

A handful of laboratory experiments have demonstrated that running on challenging surfaces such as irregular tracks or treadmills can lead to compromised postural control, including alterations in leg compression (stiffness), joint angle kinematics along changes in muscle activation (5–9). These changes in motor control, either alone or in combination, can be associated with RRIs (10, 11). However, movement control strategies during running differed depending on the perturbation modalities studied, especially regarding perturbation direction, timing, and duration (12, 13). Therefore, it is essential to assess the body's stability control mechanisms in specific perturbation scenarios to identify potential injury risks and create gait retraining programs that could potentially assist in RRI rehabilitation and/or prevention (8, 9, 14–18).

Stability control mechanisms refer to the ability to maintain an upright posture in the presence of disturbances to gait or running e.g., unstable ground. While previously utilized perturbation protocols such as changes in the ground level and pulling devices on the treadmill can yield valuable information regarding specific aspects of gait control, they are limited by the walkway length. This limitation may lead to anticipatory postural adjustments, reducing the authenticity of feedback responses as participants become aware of the perturbation location (19). Moreover, such protocols can complicate data analysis due to interfering force from obstacles or the pulling device (19–22). In contrast, split-belt treadmills offer a more effective means of simulating slip or trip-like perturbations, and provide a more suitable methodology for studying feedback responses during human gait compared to other overground and treadmill-based perturbation paradigms (23, 24).

Feedback responses are compensatory motor behaviours which can rapidly respond to changes in the body's position to maintain balance and stability, whereas feedforward responses are anticipatory motor behaviours which involve proactive adjustments that prepare the body for an upcoming movement (25–30). In recent experiments, muscle activity and kinematic adjustments of running were analysed during a trip-like perturbation protocol. The applied perturbations could elicit both feedback and feedforward neuromuscular and biomechanical responses in asymptomatic individuals (31, 32). Specifically, it has been found that muscle activity of the trunk and lower extremities muscle increased in response to perturbations in both perturbed and contralateral leg (31, 32). Furthermore, feedback responses in the perturbed leg included decreased hip adduction and stride duration, while the contralateral leg responded with reduced ankle inversion, knee and hip flexion, hip abduction, and increased stride duration and step width. Feedforward responses were only observed in the contralateral leg including decreased ankle dorsiflexion, knee flexion, hip flexion, and adduction (32). However, the findings of those investigations should be interpreted with caution due to the limited number of participants.

Both feedback and feedforward motor adaptations play an important role in motor learning, which is essential for gait retraining and injury rehabilitation programs (29, 33). Considering that adaptation depends on error feedback and the cerebellum plays a crucial role in motor adaptability, it can be argued that an internal model is responsible for controlling movement and stability (29, 34). Research has shown that older adults with a history of falls demonstrate less adaptability to repeated perturbations (35). This indicates that impairment of the cortico-cerebellar pathways with ageing can lead to reduced locomotor adaptation in older adults (29). However, evidence suggests that even a single session of perturbation training can have positive effects on both feedback and feedforward mechanisms of postural control and reduce the incidence of falls (29, 36–38).

While there have been studies investigating adaptation mechanisms for controlling posture in challenging environments during walking, there has been limited research examining these mechanisms during running. The neural control mechanisms for walking and running differ, as the muscles and joints involved in each movement require varying levels of activation and control (39). Therefore, the present study had two primary objectives: first, to examine the feedback and feedforward responses (whole-session analysis) to a perturbed running protocol, and second, to investigate the adaptation mechanisms (within-session analysis) of participants to the perturbation protocol regarding changes in kinematics and muscle activity. It was hypothesized that: (1) there would be differences in both feedback and feedforward control mechanisms of perturbed running compared to baseline measurements, and that (2) motor compensation would differ during the first encountered perturbation compared to the average of the last three perturbations.

## 2 Materials and methods

### 2.1 Participants

The study involved 23 physically active and asymptomatic individuals (9 females and 14 males), with a mean age of 29 ± 6 years, a mean weight of 68 ± 13 kg, and a mean height of 175 ± 9 cm. These individuals engaged in an average of 7 ± 4 h of physical activity per week including running, cycling, strength training, dance, soccer, rowing, extreme conditioning program training, kickboxing, hockey, and tennis. To be included in the study, participants had to meet the following criteria: aged between 18 and 50 years old, engage in physical activity for 2–20 h per week, have previous experience with running and treadmill running, and have knowledge of the English language to understand the study procedure and compliance. Exclusion criteria included having an acute infection or cold, having an acute or chronic injury in the lower extremities within the past year, having had recent surgery on the lower extremities, and having any pathology or disease that contraindicated physical activity. Participants were recruited from September 22, 2020, until July 29, 2021. All participants provided written informed consent before participating in the study, which followed the ethical guidelines of the Declaration of Helsinki and received approval from the local university Research Ethics Committee (30/2020).

### 2.2 Experimental protocol

**Figure 1:**
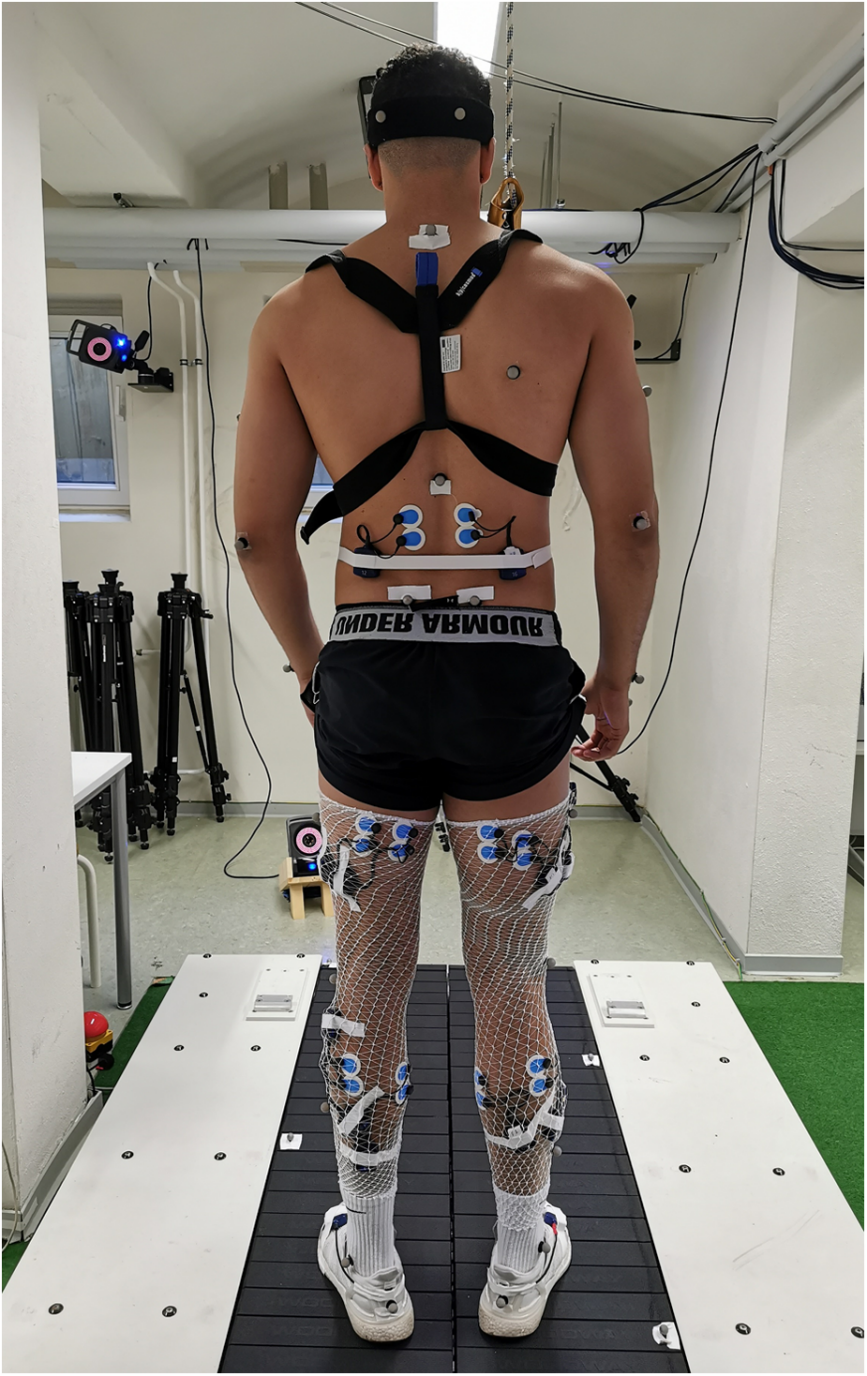
Exemplary participant and measurement set up of the split-belt treadmill. Participants were equipped with a chest harness, surface electrodes, reflective markers, and accelerometer sensors, placed on the participant's skin and shoes.

**Figure 2:**
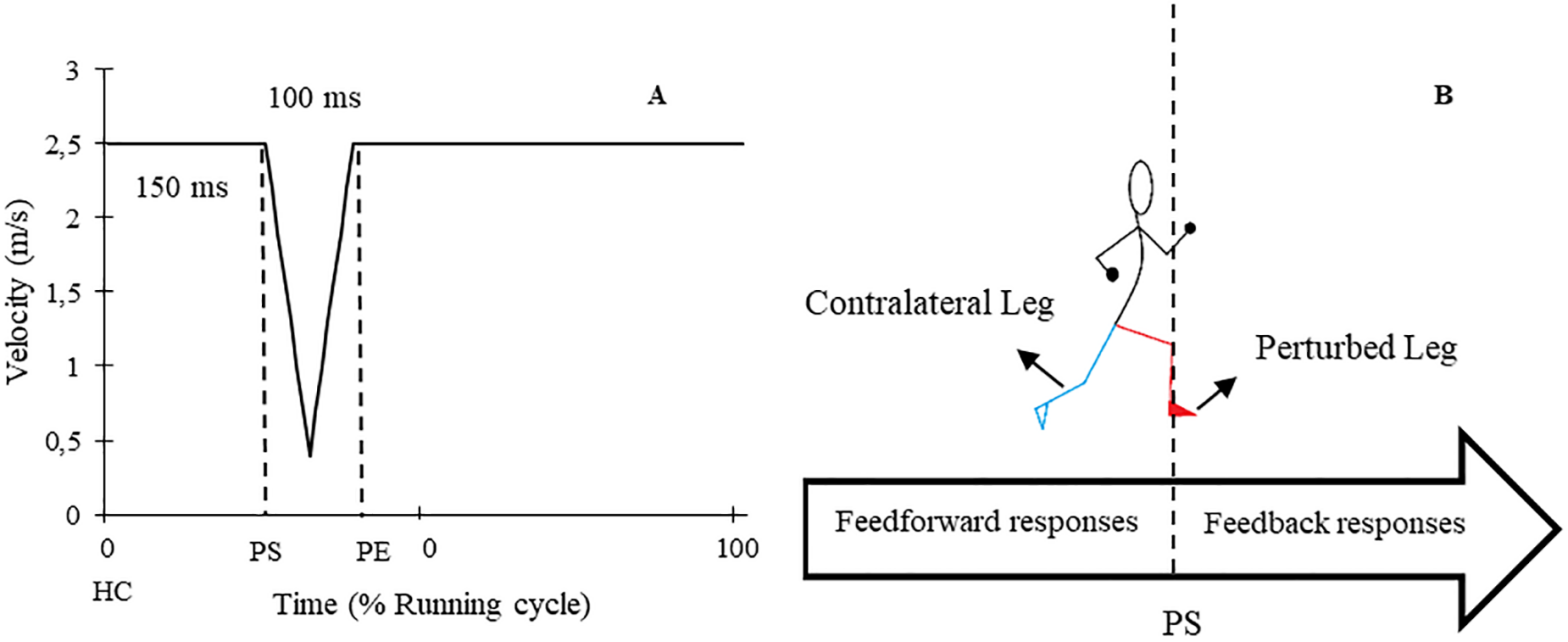
**(A)** Schematic graph of applied perturbations. **(B)** A diagram of perturbed (red) and contralateral (cyan) legs in addition to the time of feedback and feedforward responses. HC: Heel contact, PS: Perturbation start, PE: Perturbation end.

The study involved participants performing two gait assessment trials on a split-belt treadmill (Woodway, Germany) at a speed of 2.5 m/s (9 km/h) while wearing their personal shoes and being secured by a harness (see Figure 1). The first trial served as a familiarization and baseline measurement, whereby participants ran for 5 min without any perturbations. During the second trial, 30 one-sided decelerative perturbations (15 left and 15 right) were randomly superimposed on both legs, using a custom-built software (stimuli, pfitec, biomedical systems, Germany) for a period of eight minutes. Only right-sided perturbations were analysed, as right-sided perturbations were generated directly from treadmill load cells. Left-sided perturbations served to avoid unilateral running adaptations. The perturbations were initiated 150 ms after heel contact (HC), with an amplitude of 2 m/s and a duration of 100 ms (see Figure 2). Perturbations to the left side were triggered at right-heel contact and calculated by estimating step length with data taken from the baseline trial i.e., additional time delay. A minimum of 10 s elapsed between each perturbation (40). HC events were detected by an embedded load cell (Megatron, Max 5 kN, Range ± 10 mV, Soemer DAD141.1 Load cell amplifier) located beneath the right belt of the treadmill. For more detailed information on the perturbation characteristics and HC event detection method, please refer to previous studies (31, 32).

### 2.3 Instrumentation

Kinematic data were acquired using a 3D motion capture system (Vicon MX T10S, 13 cameras, 500 Hz, Vicon, Oxford, UK) and analysed using the lower body plug-in gait model (VICON Nexus 2.6) with 16 reflective markers. To record muscle activity responses, a wireless electromyography (EMG) system (4,000 Hz, Myon 320 s, Myon AG, Switzerland) was used. Bipolar round Ag/AgCl surface electrodes (2 cm inter-electrode distance, pre-gelled, Ambu, Medicotest, Denmark) were placed bilaterally on 9 muscles including erector spinae at level L2 (ES), gluteus maximus (Gmax), vastus medialis (VM), biceps femoris (BF), rectus femoris (RF), semitendinosus (ST), tibialis anterior (TA), peroneus longus (PL), and soleus (Sol). The electrodes were positioned according to SENIAM recommendations (41). Additionally, two accelerometer sensors (Myon 320 S, Myon AG, Switzerland), synchronised to the EMG data, were placed on the heel area of each participant's shoes to detect HC. A picture showing the measurement set up can be found in Figure 1.

### 2.4 Data analysis

Kinematic data underwent a zero-phase 4th order Butterworth low-pass filter with a 12 Hz cut-off frequency (Vicon Nexus 2.6). The angle curves [in degrees (°)] of the ankle, knee, and hip were analysed in all three planes (anterior-posterior, mediolateral, and rotational). HC events were identified in the kinematics data by the minimum vertical position of the heel marker (42, 43).

EMG signals [Volt, (V)] for all nine muscles were filtered using a 2nd order Butterworth low-pass filter with a 10 Hz cut-off frequency and full-wave rectified (IMAGO process master pfitec, biomedical systems, Germany). A moving average filter with a time constant of 20 ms was applied. Gait events in EMG data were determined from accelerometer sensors (42). From a previous study (32), the accelerometer sensor data was compared with the signal obtained from the load cell located beneath the treadmill belt, to evaluate the sensor accuracy. Results showed that the HC event was most accurate in the local minima of the anterior-posterior axis with a root mean square error of 12 ± 9 ms.

To evaluate kinematic and muscle activity data throughout the entire running cycle [RC, (%)]. RC was defined from HC prior to perturbation onset until HC of the same leg after the perturbation. The joint angle and muscle activity curves were normalized to 101 points. This normalization allowed for continuous assessment of the data during all phases of the stride cycle. To analyse the responses and adaptations to perturbations, both feedback and feedforward responses were examined in the kinematic and muscle activity curves. Feedforward responses occurred from the HC event until the initiation of the perturbation whereas, feedback responses referred to the changes after the perturbation onset until the next HC event on the same leg.

To evaluate the whole-session responses to the perturbations, the EMG amplitude and joint angle curves of 10 consecutive strides from the end of the baseline trial for each participant were averaged (44–46), and this was compared to the average of all perturbed strides from the perturbed running trial. To assess within-session adaptation over the course of the 8-minute perturbed running protocol, the first encountered right-sided perturbation was compared to the average of the last three perturbations on the same side (46). The assessment of responses and adaptations was performed on both the perturbed leg (PertL, right side) and contralateral swing leg (ContraL, left side) during right-sided perturbations (see Figure 2) (28, 32, 47). Perturbed strides were included when changes in the position of the heel marker in kinematic data or the signal of the accelerometer data were visible.

### 2.5 Statistical analysis

Data were analysed with one-dimensional statistical parametric mapping (SPM) of Paired *T*-tests to assess responses and adaptations to the perturbations. One-dimensional SPM is a statistical analysis technique that involves the continuous comparison of multiple field observations along a single dimension (usually time) to identify significant changes in activity related to a specific task. The open-source spm1d package [v. 0.4, https://www.spm1d.org, (48)] was used to test joint angle and muscle activity curves (MATLAB®, R2020a, MathWorks Inc., Natick, MA, USA). Supra-threshold clusters were built to indicate a significant difference between the unperturbed and perturbed trial for whole-session response analysis, as well as the first and the average of the last three perturbations for within-session adaptation analysis. If the SPM{t} trajectory crossed the critical threshold at any time node, the null hypothesis was rejected. To retain a family-wise Type 1 error rate of α = 0.05, a Bonferroni corrected threshold of 0.025 for two sets of comparisons (PertL and ContraL) was applied.

## 3 Results

### 3.1 Feedback and feedforward whole-session responses

#### 3.1.1 Kinematics responses

**Figure 3:**
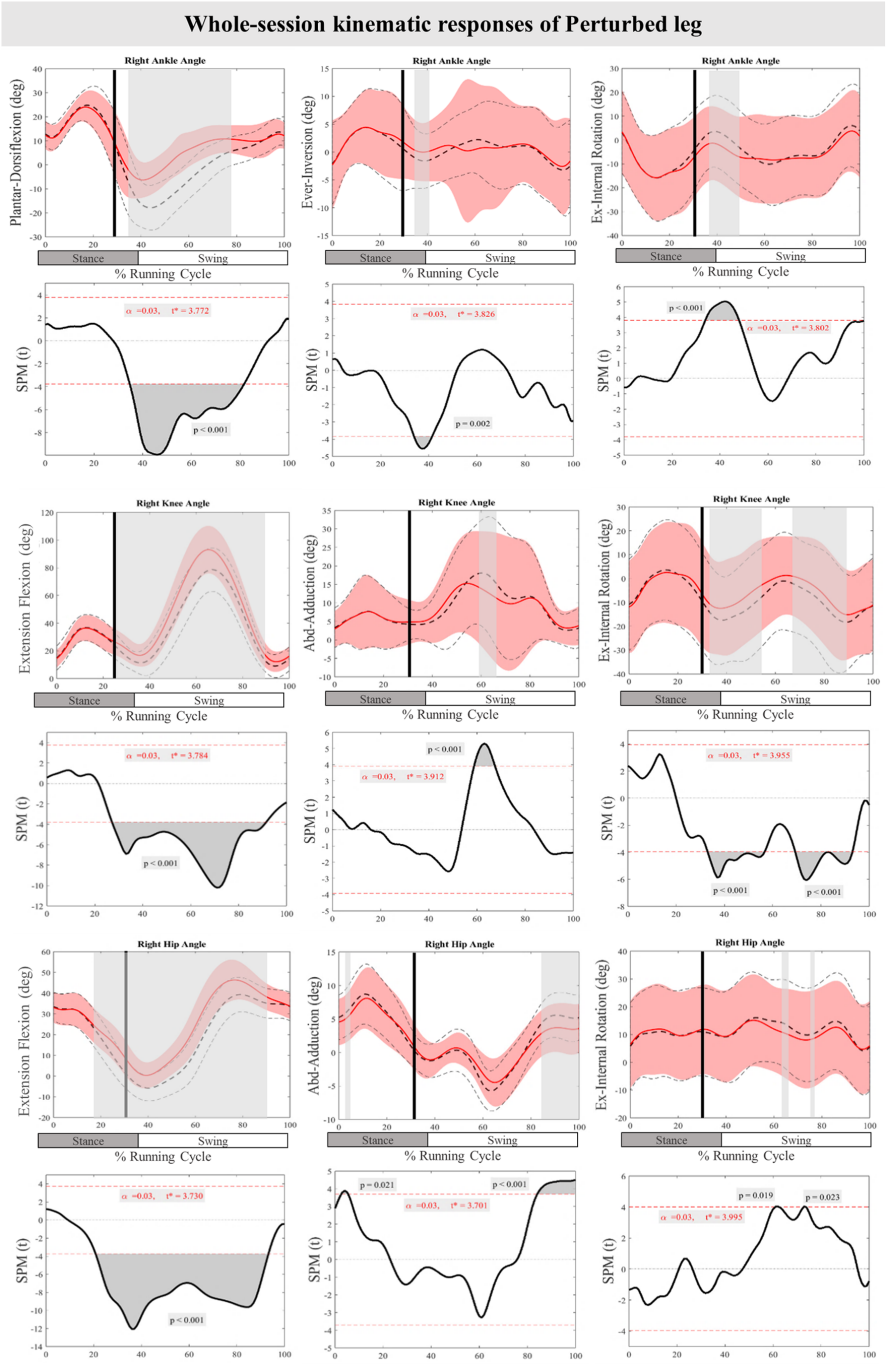
Mean and SD curves of joint angle responses for the perturbed leg. Running cycle data of the baseline (black dashed line) and perturbed (red line) running, as well as time-dependent t-values of the SPM are pictured. The vertical black line signals the onset of perturbation. Grey bars designate the areas where the critical threshold was exceeded, an indication of statistically significant deformation of the angle curves in response to the perturbations (*P* < 0.025). Positive values represent dorsiflexion, inversion, flexion, adduction, and internal rotation movements.

Figure 3 presents the mean and standard deviation (SD) curves of the joint angle responses and time-dependent t-values of the SPM for the PertL. The results showed significant changes in feedback responses of the perturbed trial in all joint angles of the PertL when compared to the baseline trial. Supra-threshold clusters showed significant differences in the ankle joint including decreased plantarflexion at 35%–82%, decreased eversion at 34%–41% and decreased internal rotation at 34%–48% of the RC. Random curves of identical smoothness would produce these results with a probability of *P* < 0.001, *P* = 0.002, and *P* < 0.001, respectively. The study found increased knee flexion at 30%–91%, decreased knee adduction at 56%–67%, and decreased knee external rotation at 33%–56% & 70%–93% of the RC in response to perturbations, all with a probability of *P* < 0.001. The hip joint showed decreased extension at 30%–94% (*P* < 0.001), decreased adduction at 85%–100% (*P* < 0.001) and decreased internal rotation movement at 60%–63% & 73%–75% (*P* = 0.019 & *P* = 0.023) of the RC. Furthermore, the study found participants' feedforward response included decreased hip extension at 21%–30% (*P* < 0.001) and decreased hip adduction at 3%–5% (*P* = 0.021) of the RC (see Figure 3).

**Figure 4:**
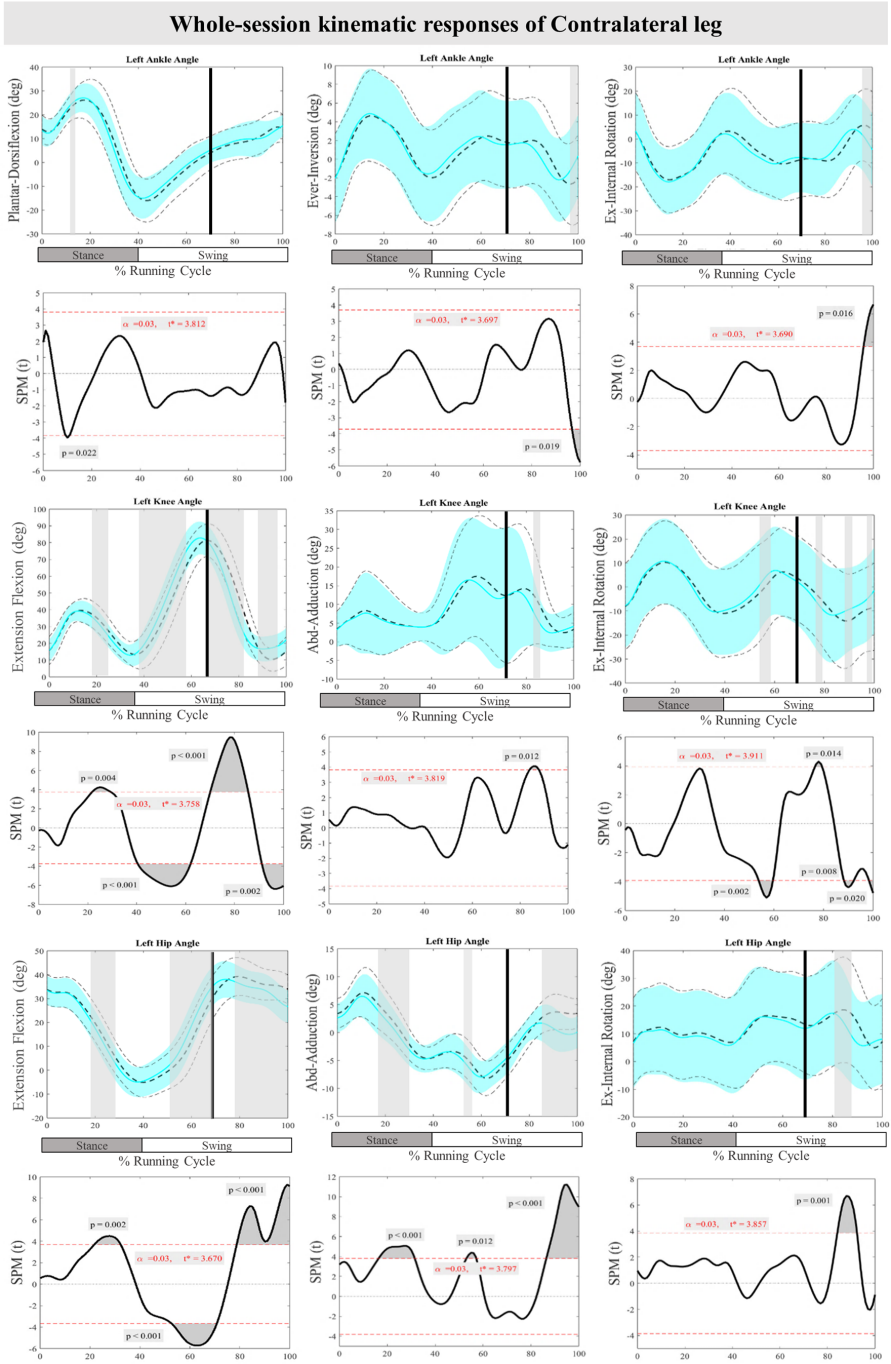
Mean and SD curves of joint angle responses for the contralateral leg. Running cycle data of the baseline (black dashed line) and perturbed (cyan line) running, as well as time-dependent t-values of the SPM are pictured.

Figure 4 displays the mean and SD curves for the joint angle responses, as well as the time-dependent t-values of the SPM for the ContraL. The perturbations applied during the study caused certain feedback adjustments in the ContraL, such as decreased ankle inversion at 96%–100% (*P* = 0.019) and ankle internal rotation at 96%–100% (*P* = 0.016). Additionally, the participants made adjustments in the angle of both knee and hip joints of ContraL in all three planes. Specific adjustments included decreased knee flexion after perturbation at 70%–85% (*P* < 0.001), increased knee flexion at 91%–100% (*P* = 0.002), decreased knee adduction at 84%–87% (*P* = 0.012), increased knee external rotation at 76%–80% (*P* = 0.014) and decreased knee external rotation at 88%–92% & 98%–100% (*P* = 0.008 & *P* = 0.020) of the RC. The hip joint also showed significant changes, with decreased hip flexion movement at 79%–100% (*P* < 0.001) as well as decreased hip adduction at 86%–100% (*P* < 0.001) and hip internal rotation at 84%–92% (*P* < 0.001) of the RC. For the ContraL, feedforward responses to the perturbations included increased ankle dorsiflexion at 10%–11% (*P* = 0.022), decreased knee flexion at 22%–28% (*P* = 0.004) and increased knee flexion at 40%–62% (*P* < 0.001), increased knee internal rotation at 54%–60% (*P* = 0.002), increased hip extension at 22%–32% (*P* = 0.002), increased hip flexion at 53%–70% (*P* < 0.001), and decreased hip adduction at 16%–31% & 54%–57% (*P* < 0.001 & *P* = 0.012) of the RC in comparison to baseline. Figure 4 provides a visual representation of these findings.

#### 3.1.2 Muscle activity responses

**Figure 5:**
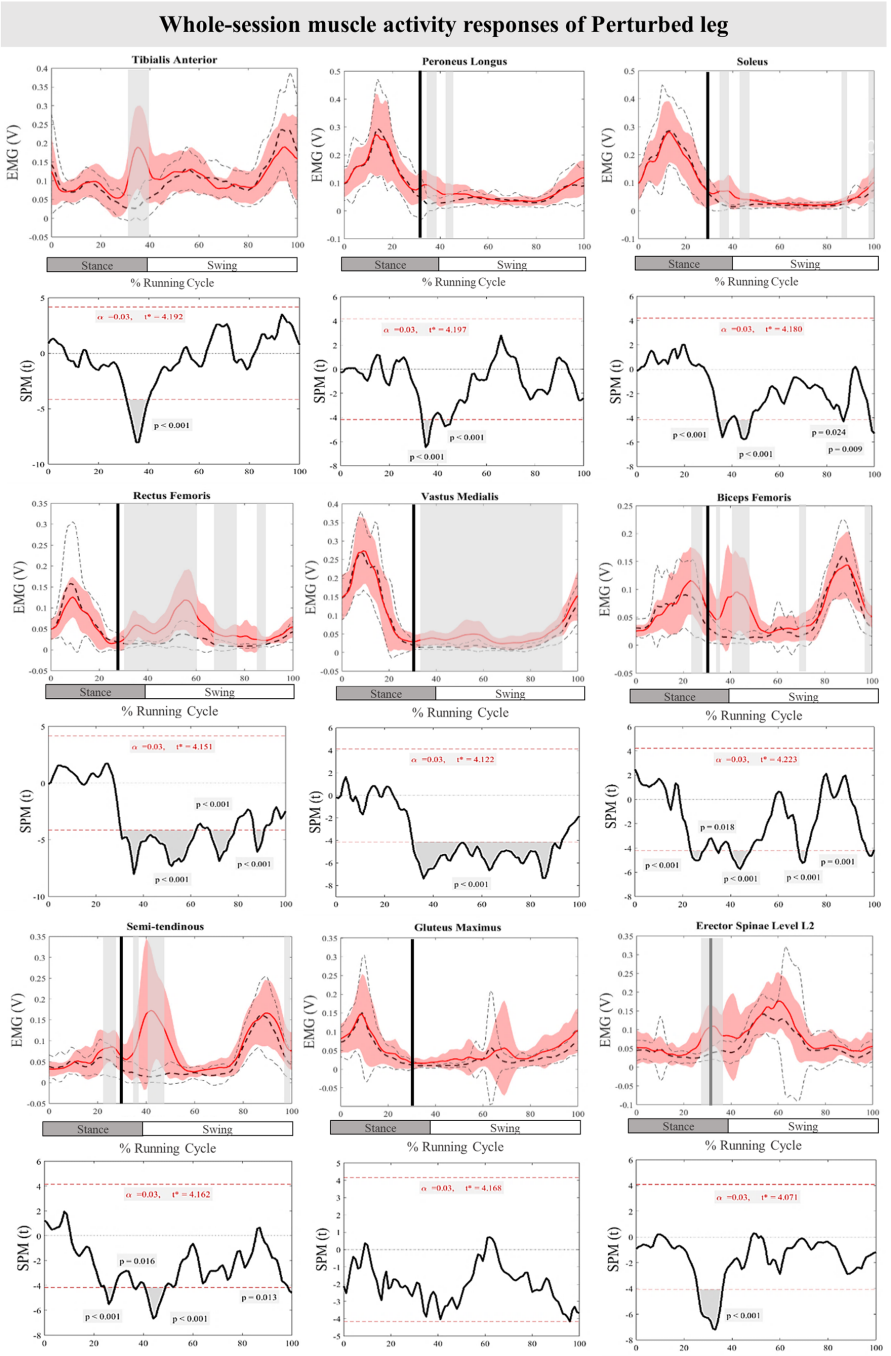
Mean and SD curves of muscle activity responses for the perturbed leg. Running cycle data of the baseline (black dashed line) and perturbed (red line) running, as well as time-dependent t-values of the SPM are pictured.

The curves of the muscle activity responses and time-dependent t-values of the SPM for the PertL are depicted in Figure 5. Supra-threshold clusters were significant in all measured muscles in feedback responses except for Gmax when compared to the baseline trial. Altered muscle activity in lower leg muscles included increased activity of TA at 31%–40% (*P* < 0.001), PL at 34%–38% & 42%–45% (*P* < 0.001) and Sol at 35%–49%, 42%–48%, 86%–87% and 98%–100% (*P* < 0.001, *P* < 0.001, *P* = 0.024, & *P* = 0.009) of the RC. The activity of BF at 34%–36%, 40%–49%, 69%–72%, & 97%–100% (*P* = 0.018, *P* < 0.001, *P* < 0.001, & *P* = 0.001), VM at 31%–93% (*P* < 0.001), RF at 30%–62%, 67%–78% & 87%–91% (*P* < 0.001), ST at 36%–37%, 41%–49%, & 98%–100% (*P* = 0.016, *P* < 0.001, & *P* = 0.013), and ES at 30%–35% (*P* < 0.001) also increased significantly in different phases of the RC. Feedforward responses to the perturbations included increased BF and ST muscle activity at 23%–29% and 25%–29% (*P* < 0.001) of the RC, respectively, as well as ES at 26%–30% (*P* < 0.001) of the RC (Figure 5).

**Figure 6:**
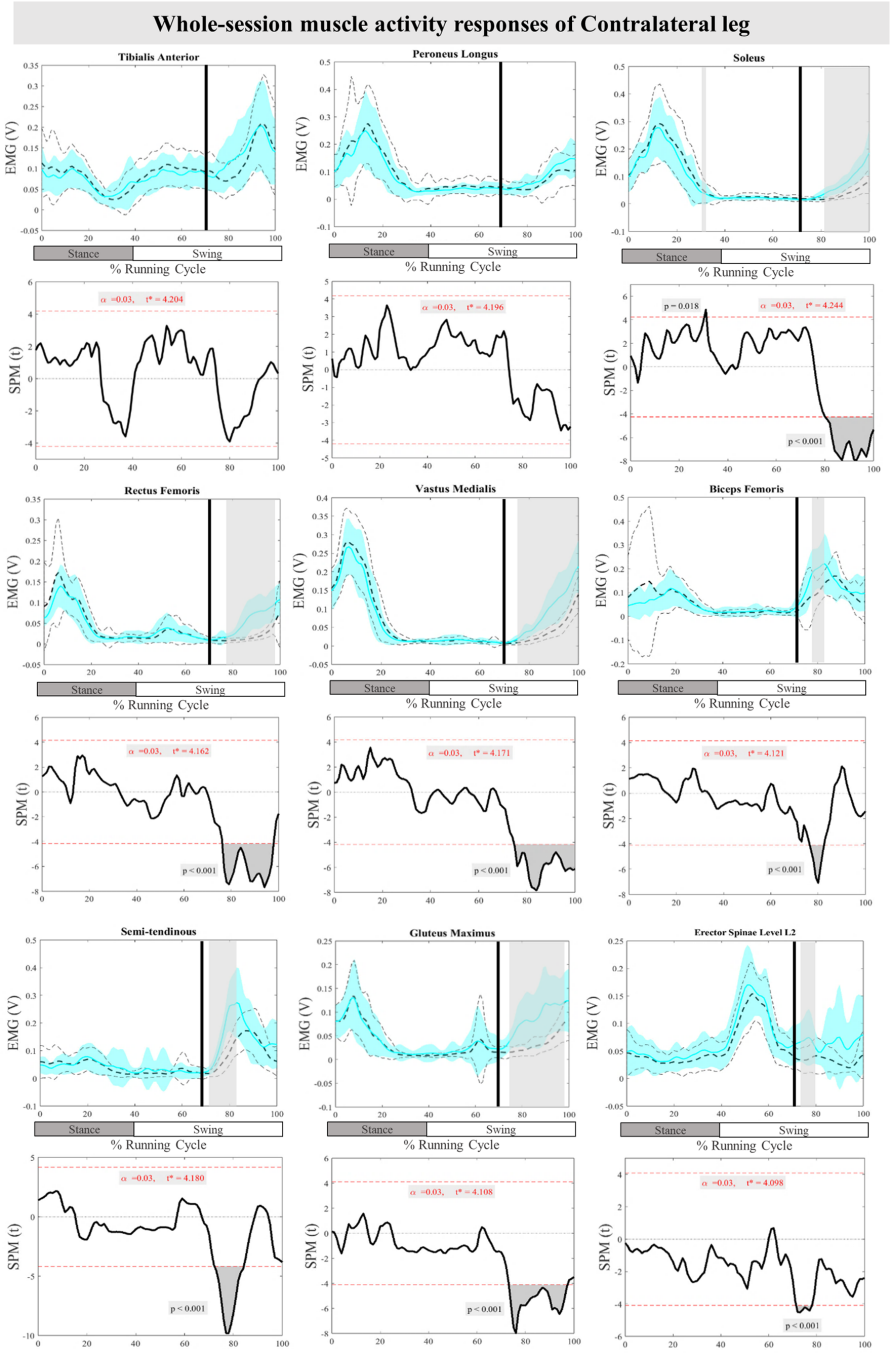
Mean and SD curves of muscle activity responses for the contralateral leg. Running cycle data of the baseline (black dashed line) and perturbed (cyan line) running, as well as time-dependent t-values of the SPM are pictured.

The mean and SD curves of the muscle activity responses and time-dependent t-values of the SPM for the ContraL are presented in Figure 6. Significant differences in feedback muscle activities were observed in all measured muscles during the perturbation trial except for TA and PL in comparison to baseline. Supra-threshold clusters exceeded the critical threshold, in Sol at 80%–100% (*P* < 0.001), RF at 76%–98% (*P* < 0.001), VM at 75%–100% (*P* < 0.001), BF at 77%–82% (*P* < 0.001), ST at 72%–85% (*P* < 0.001), Gmax at 73%–97% (*P* < 0.001), and ES at 71%–79% (*P* < 0.001) of the RC, indicating statistically significant results. Additionally, feedforward responses were observed in Sol at 30%–31% (*P* = 0.018) (Figure 6).

### 3.2 Feedback and feedforward within-session adaptations

#### 3.2.1 Kinematic adaptations

**Figure 7:**
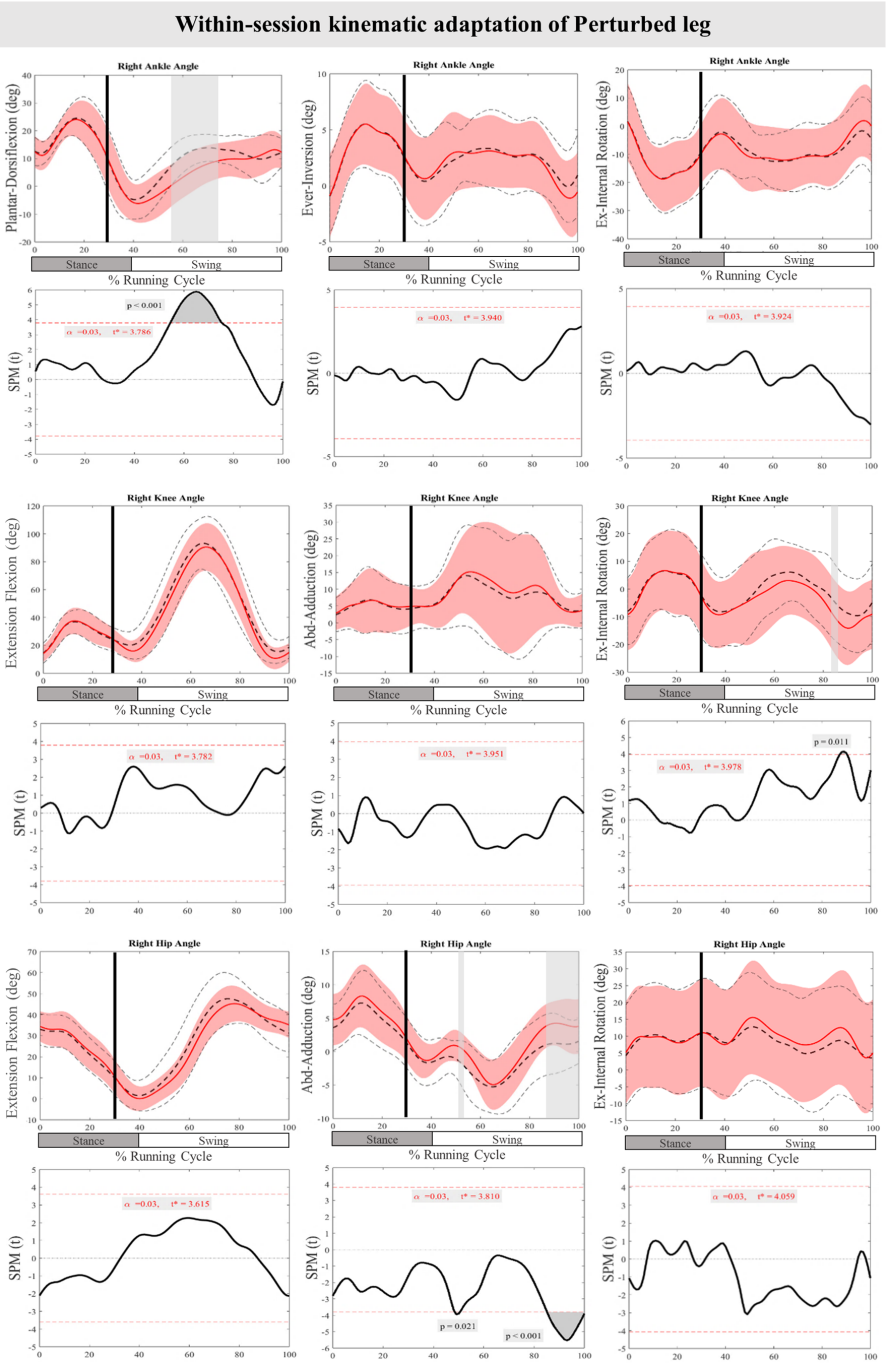
Mean and SD curves of joint angle adaptations for the perturbed leg. Running cycle data of the first (black dashed line) and the last three (red line) perturbations, as well as time-dependent t-values of the SPM are pictured.

Figure 7 presents the mean and SD curves of the joint angle feedback adaptations and time-dependent t-values of the SPM for the PertL. SPM analysis of the last three perturbations showed a statistically significant decrease in ankle dorsiflexion at 55%–75% of the RC (*P* < 0.001), an increase in knee external rotation at 87%–90% of the RC (*P* = 0.011) and an increase in hip adduction at 43%–50% & 85%–100% of the RC (*P* = 0.021 & *P* < 0.001). Regarding feedforward adaptations, no statistically significant differences were found between the first and the average of the last three perturbations in joint angles of the PertL (Figure 7).

**Figure 8:**
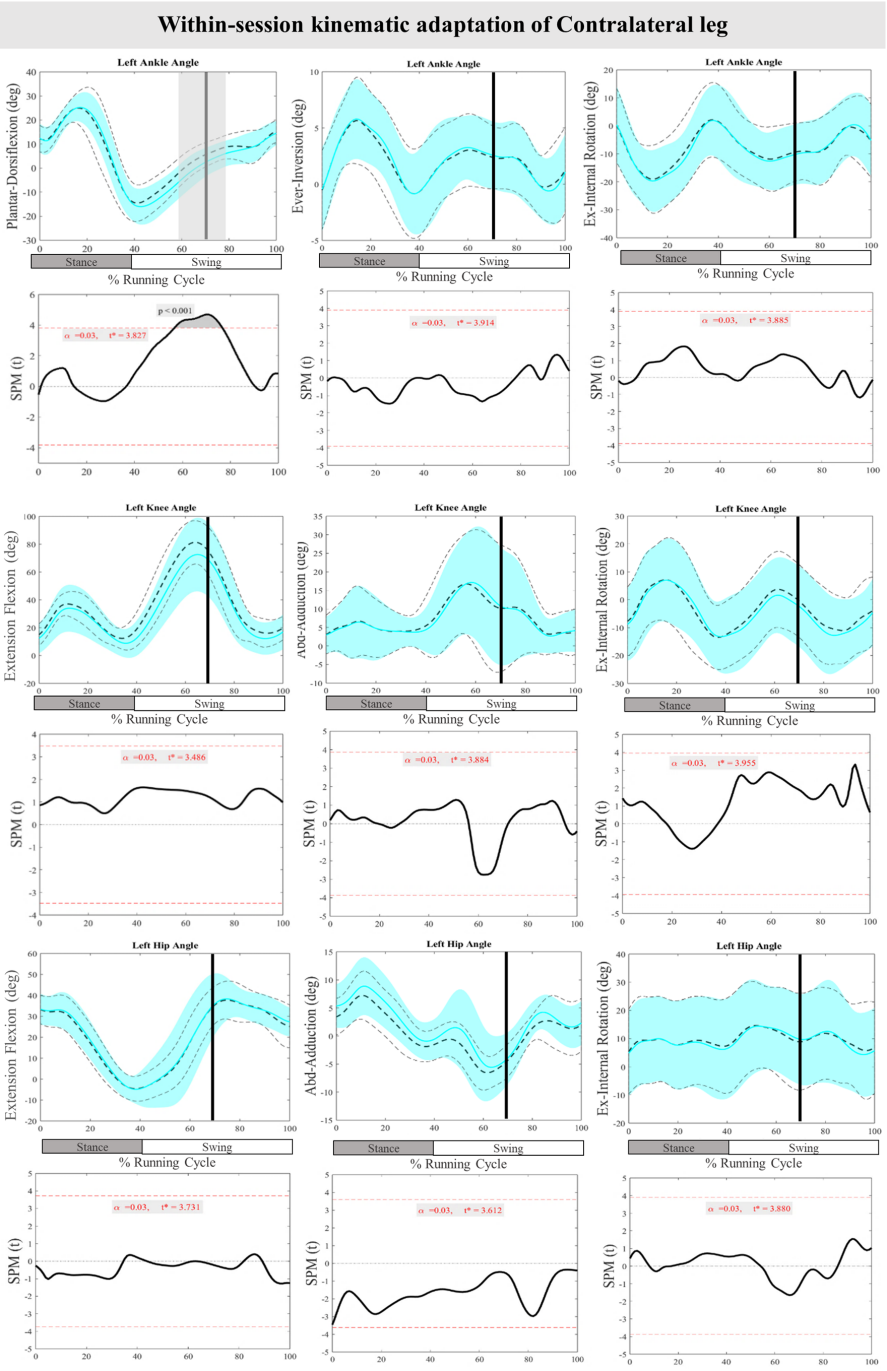
Mean and SD curves of joint angle adaptations for the contralateral leg. Running cycle data of the first (black dashed line) and the last three (cyan line) perturbations, as well as time-dependent t-values of the SPM are pictured.

Figure 8 presents the mean and SD curves of the joint angle adaptations and time-dependent t-values of the SPM for the ContraL. Perturbations caused an adaptation only in ankle movement, with decreased dorsiflexion at 70%–76% of the RC (*P* < 0.001). Additionally, decreased dorsiflexion of the ankle at 57%–70% of the RC (*P* < 0.001) was observed in preparation for the perturbations in the ContraL (Figure 8).

#### 3.2.2 Muscle activity adaptations

**Figure 9:**
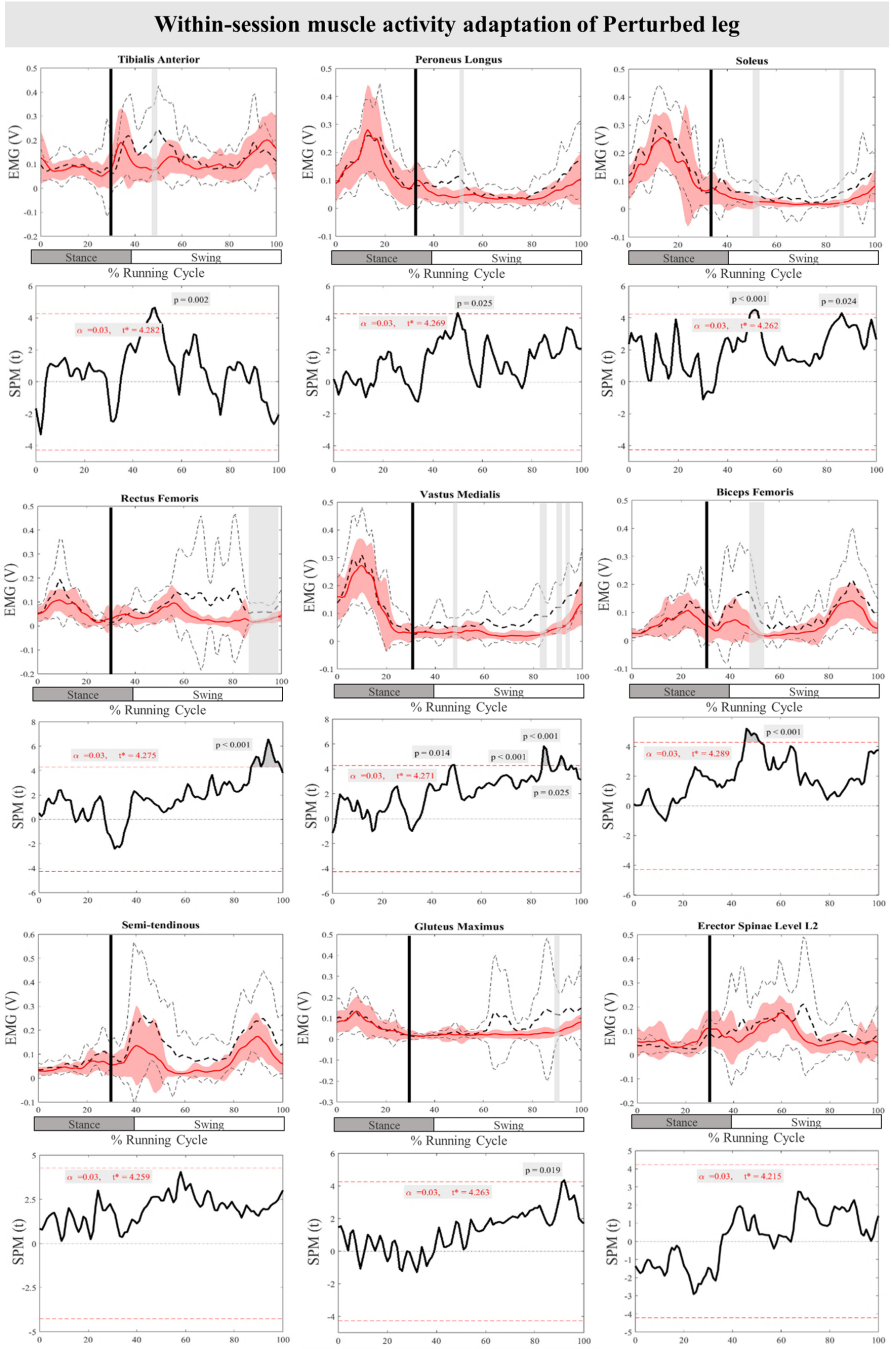
Mean and SD curves of muscle activity adaptations for the perturbed leg. Running cycle data of the first (black dashed line) and the last three (red line) perturbations, as well as time-dependent t-values of the SPM are pictured.

The mean and SD curves of the muscle activity adaptations and time-dependent t-values of the SPM for the PertL are presented in Figure 9. Altered feedback adaptation in muscle activity of the lower leg muscles was observed in the average of the last three perturbations, including the decreased activity of TA at 47%–50% (*P* = 0.002), PL at 50%–51% (*P* = 0.025), and Sol 50%–52% & 85%–86% (*P* < 0.001 & *P* = 0.024) of the RC. Moreover, the activity of upper leg muscles RF at 87%–99% of the RC (*P* < 0.001), VM 47%–49%, 85%–88%, 90%–94%, & 95%–96% (*P* = 0.014, *P* < 0.001, *P* < 0.001, & *P* = 0.025), and BF at 45%–52% (*P* < 0.001) of the RC also decreased. The activity of the Gmax at 91%–92% (*P* = 0.019) of the RC was also reduced for the average of the last three perturbations. No statistically significant difference was detected in muscle activity feedforward adaptations of the PertL leg (Figure 9).

**Figure 10:**
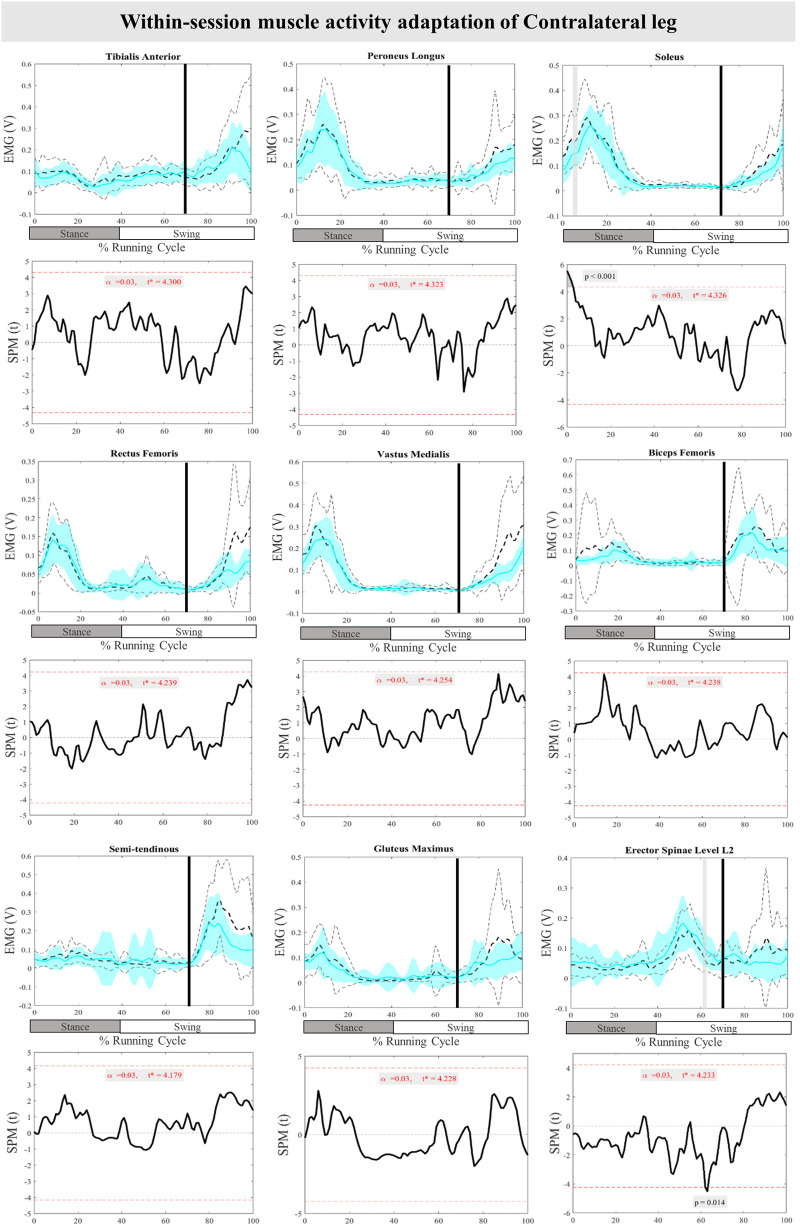
Mean and SD curves of muscle activity adaptations for the contralateral leg. Running cycle data of the first (black dashed line) and the last three (cyan line) perturbations, as well as time-dependent t-values of the SPM are pictured.

The mean and SD curves of the muscle activity adaptations and time-dependent t-values of the SPM for the ContraL are presented in Figure 10. No significant adaptations and time-dependent t-values of the SPM in feedback adaptation in muscle activity data of the ContraL leg were observed when the average of the last three perturbations was compared to the first perturbation. Limited feedforward adaptation was noticed in the muscle activity of the ContraL leg including decreased Sol at 0%–3% (*P* < 0.001), and increased ES at 62%–64% (*P* = 0.014) of the RC muscle activity (Figure 10).

## 4 Discussion

The primary aim of the present study was to investigate the feedback and feedforward mechanisms involved in postural control during running when subjected to a trip-like perturbation among asymptomatic individuals. Participants experienced perturbations during the early stance phase of running and joint angle curves and EMG amplitudes of nine different muscles of the lower body and trunk in both the perturbed and contralateral legs were analysed. The findings confirm the first part of our hypothesis, indicating that perturbations indeed induced changes in both feedback and feedforward control mechanisms of running compared to the baseline measurement. However, the second hypothesis, which suggested greater muscle activity and movement modification during initial perturbation compared to the average of the last three perturbations, was only partially supported. Consequently, within-session adaptation was mostly observed in the feedback mechanisms of the perturbed leg with a limited adaptation of feedforward mechanisms.

### 4.1 Whole-session responses

In the current protocol, immediately following the perturbation (at 30% of the RC), the perturbation caused a backward movement of the right leg, placing participants in an unstable position, which can lead to an increased trunk flexion angle. This instability is reflected in the increased activity of the ES muscles post-perturbation. To compensate for the backward leg movement, participants increase the activity of the TA to lift the foot off the ground, accompanied by increased knee and hip flexion to move the leg forward and counteract the loss of forward momentum. The contralateral leg also supports this movement by increasing the activity of the soleus and upper leg muscles to ensure a stable landing at the time of second HC. This is in line with previous studies assessing the kinetics and kinematics of the leg during running across a visible and camouflaged 10 cm drop on level ground (6, 8) where knee flexion angle increased in both visible and camouflaged 10 cm drop scenarios. Furthermore, perturbations in this study affected ankle, knee and hip movement in the frontal and horizontal planes, however, these changes occurred briefly during the gait cycle. In the frontal plane, decreased inversion of the ankle joint at the time of toe-off was also observed, whichcan shift the force to the medial part of the foot and potentially increase the risk of patella femoral pain syndrome (49).

In terms of muscle activity, significant but brief increases in the activity of TA, Pl, and Sol muscles after the initiation of perturbation were recorded. Thereafter, the activity of lower leg muscles was silent during the swing phase of running which was also reported in the study of a mechanical perturbation specifically targeting the swing phase of running (50). It has been argued that these muscles do not have a role during the swing phase of the running cycle (50). Meanwhile, the muscle activity of the Sol muscle rose again at the time of the heel strike in both the present study and Scohier et al. (50). Notably, the VM and RF muscles were activated throughout most of the runing cycle in response to the perturbations, except toward the end of it. Additionally, the hamstring muscles (BF & ST) showed significant activation immediately after perturbation and toward the end of the running cycle. This selective timing of leg muscle activation has been suggested to shift the body's control strategy from precise movement to a more robust approach, enhancing the ability to cope with errors during unexpected perturbations (51).

In the present study, unilateral perturbations led to bilateral responses in both the displaced and non-displaced sides. In the ContraL, most alterations occurred towards the end of the RC, with the ankle plantarflexion angle remaining unchanged compared to the unperturbed trial, consistent with our previous study of perturbed running with the current protocol but based on a smaller sample size (32). While TA and PL muscles of the ContraL did not compensate for the perturbations, Sol, RF, VM, BF, ST, Gmax, and ES muscle activity increased. This aligns with prior studies highlighting the importance of ContraL in successfully recovering from perturbations during walking (52–54). Conversely, studies on walking perturbations have emphasized the role of distal lower leg muscles, including TA and Gastrocnemius, in compensating for disturbances (52) while findings of the current study suggest a more important role for the proximal segment.

Participants in our experiment exhibited increased pre-activity of BF, ST, ES of PertL as well as Sol muscle of ContraL in anticipation of upcoming perturbations. These responses are indicative of previous research on camouflage drops in level ground during running (16) and various studies during perturbed walking (55–59). This study did not investigate trunk movement modifications, however, increased activity of the ES muscle underscores the significant role of the trunk muscles in compensatory and anticipatory control of upright posture during perturbed running. In the study of van der Burg et al. trunk movement and muscle activity of ES at the level of L1 and T9 increased after a trip perturbation during walking (60).

Interestingly, our study found simultaneous recruitment of both lower leg muscles (TA and Sol) and upper leg muscles (VM and BF) in response to perturbations, differing from previous research on perturbed walking (12, 61), where lower leg muscles were activated earlier than thigh muscles. The activation of both agonist and antagonist muscles (BF and ST) increased in response to perturbations, similar to the findings of camouflaged drops (16), where both the TA and gastrocnemius muscles increased in activity. This suggests a high degree of co-contraction, characterized by the overlap of agonist and antagonist muscle activity.

### 4.2 Within-session adaptations

Besides comprehending the core stability control mechanisms when responding to perturbations, a key question arises: Can participants adapt within a single session of perturbed running? Prior research has illustrated that extended exposure to perturbations during walking can enhance motor adjustments in both younger and older adults over time (29, 36, 55, 59). Nevertheless, studies have shown that these adaptations can manifest within a single session of perturbation training (62). In this study, adaptations were predominantly evident in feedback responses, characterized by a significant decrease in dorsiflexion of the ankle, increased knee external rotation, and an increase in hip adduction of the PertL. Moreover, muscle activity in the TA, PL, Sol, RF, VM, BF, and Gmax decreased in the average of the last three perturbations compared to the initial perturbation. Unfortunately, there is a scarcity of studies exploring feedback adaptation in response to perturbation, with some studies showing improvement in step length and stability (29, 51).

Notable feedforward adaptations were observed in joint angles and muscle activity of the PertL. Karamanidis et al. and Chambers and Cham, have also respectively reported an increased base of support while walking against an external resistance and heightened muscle activity during adaptation to slip perturbations of the PertL (63, 64). Moreover, a separate study examining adaptation mechanisms to slip perturbations during walking found that feedback control of stability improved 17% more than feedforward control of stability (65). Regarding the ContraL, plantarflexion of the ankle joint increased during both feedback and feedforward adaptations. However, while muscle activity showed no significant changes during feedback adaptations, feedforward adaptation led to decreased Sol and increased ES muscle activity. These results align with those reported in the study by Hsu et al., where plantar flexion of the ContraL increased during stepping onto a moving surface (66).

Participants adapted their muscle activity and joint angles at the end of the protocol in comparison to the initial novel perturbation. These adaptations occurred at the time of toe off and in preparation for the next foot contact. This process reflects motor learning, where the nervous system refines its control strategies based on repeated exposure to perturbations (67, 68). Motor learning involves the continuous updating of the internal model framework, which includesboth forward and inverse models, to adapt to perturbations during walking and running (29, 33). This framework allows the CNS to predict the consequences of motor commands and adjust them based on sensory feedback, ensuring smooth and stable movement in the face of unexpected changes (68, 69). Therefore, current results suggest that the nervous system shifts from a more robust control strategy to a more precise and accurate movement control in response to repeated perturbations (51, 67). Studies proposed that the cerebellum supports the continuation of the movement by updating and predicting error correction. In other words, the cerebellum uses this error feedback to refine the inverse and feedforward models, making movements more accurate over time (67–69).

### 4.3 Limitations

Presently, there are no published reports concerning feedback and feedforward adaptation to stumbling during running, whether within a single session or over the long term. The contradictory findings, combined with the limited research in this area, emphasize the necessity for future studies investigating various types of perturbation modalities during running. Future studies should also examine trunk movement, given that sudden and unforeseeable loading remains a significant contributor to low-back pain (28). In this study, we compared the baseline trial with all perturbations, as well as the first perturbation with the last three perturbations. This approach may limit our understanding of how the initial perturbation affects the regular running pattern and how adaptations occur with repeated exposure. Future studies could refine this approach by comparing the baseline trial solely with the first perturbation to assess the immediate impact and separately comparing the baseline with the last three perturbations to examine acute changes in running patterns following repeated perturbations. It is also important to examine adaptation immediately following the first perturbation, as research has demonstrated that feedforward locomotor adaptation occurs rapidly and continues to improve over time (29). The current experiment did not evaluate the retention of adaptation to the applied perturbations, which could be crucial since the effectiveness of the perturbation protocol relies on both rapid adaptation and ensuring long-term retention (36). The study examined only younger adults, however, future studies should include older adults as well, as previous studies have indicated a deficiency in feedback adaptation among this demographic in response to slip perturbations (29, 58). The frontal and horizontal plane movements often involve complex joint movements, particularly at the knee, hip, and pelvis. The simplified joint models in PlugInGait may not fully capture these complexities (70). A limitation of the designed treadmill perturbation protocol is its inability to fully replicate real-world conditions. However, the current treadmill-based perturbation protocol produces whole-body responses, enabling the study of physiological reactions to stumbling (31, 71).

### 4.4 Conclusion

The findings suggest a significant reaction characterized by an immediate increase in muscle activity following applied perturbations, potentially aimed at stabilizing the body's equilibrium. While lower leg muscles exhibited momentary activation in response to perturbations, upper leg muscles showed sustained activation throughout the entire running cycle. This implies a more centralized approach to stability control during running when compared to walking. Overall, it appears that the body employs movement adjustments and increased muscle activity of the perturbed leg to protect itself from falling while simultaneously preparing for the next heel strike, thus averting instability or injury. The within-session adaptation to perturbations during running, particularly in the reactive responses of the perturbed leg, suggests acute motor learning. This implies that the current protocol could be valuable for training purposes within rehabilitation programs or when challenging terrain for training is not readily available.

## Data Availability

The raw data supporting the conclusions of this article will be made available by the authors, without undue reservation.
